# Supplementary material for: IRE1-mediated degradation of pre-miR-301a promotes apoptosis through upregulation of GADD45A
Source: Cell Commun Signal. 2023 Nov 9;21:322. doi: 10.1186/s12964-023-01349-0 (PMC10634081; doi:10.1186/s12964-023-01349-0)
Supplement: Supplementary file 2 — Additional file 1. [file 12964_2023_1349_MOESM1_ESM.pdf]

Supplemental Data

IRE1-mediated degradation of *pre-miR-301a* promotes apoptosis through upregulation of *GADD45A*

Magdalena Gebert[1], Sylwia Bartoszewska[2], Lukasz Opalinski[3], James F. Collawn[4] and Rafał Bartoszewski[5*]

[1]Department of Medical Laboratory Diagnostics – Fahrenheit Biobank BBMRI.pl, Medical University of Gdansk, Gdansk, Poland.

[2]Department of Inorganic Chemistry, Medical University of Gdansk, Gdansk, Poland.

[3]Department of Protein Engineering, Faculty of Biotechnology, University of Wroclaw, Wrocław, Poland

[4]Department of Cell, Developmental, and Integrative Biology, University of Alabama at Birmingham, Birmingham, USA, Birmingham, AL 35294.

[5]Department of Biophysics, Faculty of Biotechnology, University of Wrocław, F. Joliot-Curie 14a Street, 50-383 Wrocław, Poland; rafal.bartoszewski@uwr.edu.pl

*****Correspondence: Rafał Bartoszewski, Department of Biophysics, Faculty of Biotechnology, University of Wrocław, F. Joliot-Curie 14a Street, 50-383 Wrocław, Poland; rafal.bartoszewski@uwr.edu.pl

Supplemental Figure 1

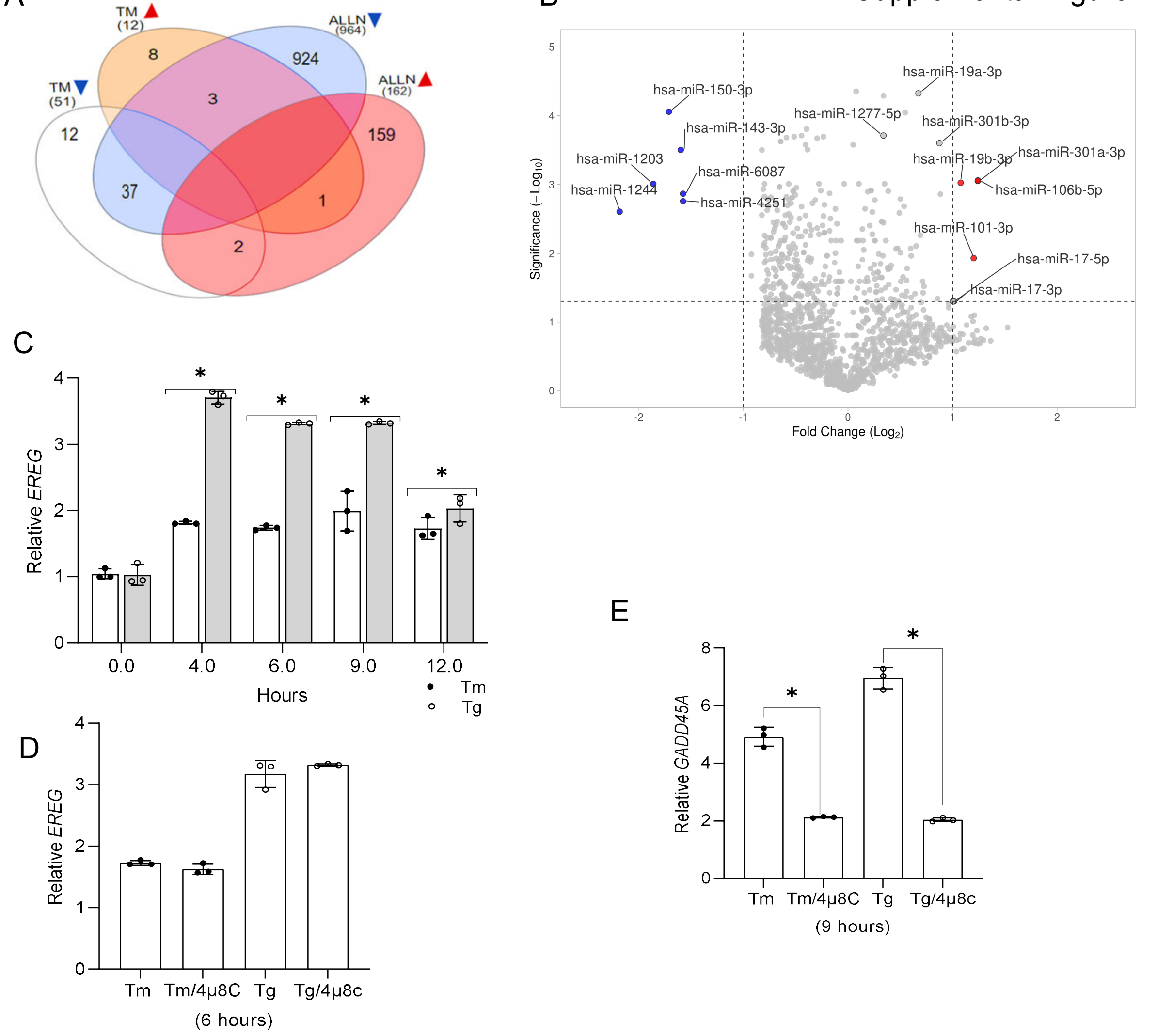

Supplemental Figure 1. The Venn diagram (62) **represents the general distribution of miRNAs that were significantly affected by IRE1 inhibition during ALLN and Tm induced ER stress (A).** MiRNA reduced in Tm treated 16HBE14o- cells in the presence of 4μ8C are in white the eclipse, whereas upregulated miRNAs are in light orange. MiRNA reduced in ALLN treated 16HBE14o- cells in the presence of 4μ8C are in blue eclipse, whereas upregulated miRNAs are in red. 4μ8C at 20μM concentration, Tm at 2.5μg/ml, and ALLN at 100 μM were incubated for 6 hours. (**B**) The Volcano plot represents the NGS profiling results of changes in miRNA expression in 16HBE14o- cells exposed to in tunicamycin (Tm, 2.5 μg/ml) in the presence of the IRE1 inhibitor 4μ8C. 4μ8C was used at 20 μM concentration. A P value of $P < 0.05$ was considered significant. ER stress-induced changes in *EREG* mRNA levels in 16HBE14o- cells for 12 hours and the impact of 4μ8C on *EREG* expression (**D** and **E**) were analyzed by qRT-PCR and normalized to *RPLP0* mRNA levels and expressed as a fold change over to the no-stress samples at 6 and 9 hours. The results from three independent experiments ($n = 9$) are plotted and expressed as a fold change over the no-stress controls. Error bars represent standard deviations. Significant changes (P value $P < 0.05$) are marked with an asterisk. ER stressors used: Tm (2.5 μg/ml), Tg (50 nM)).

(62) Heberle, H., Meirelles, G. V., da Silva, F. R., Telles, G. P., and Minghim, R. (2015) InteractiVenn: a web-based tool for the analysis of sets through Venn diagrams. *Bmc Bioinformatics* **16**

Supplemental Figure 2

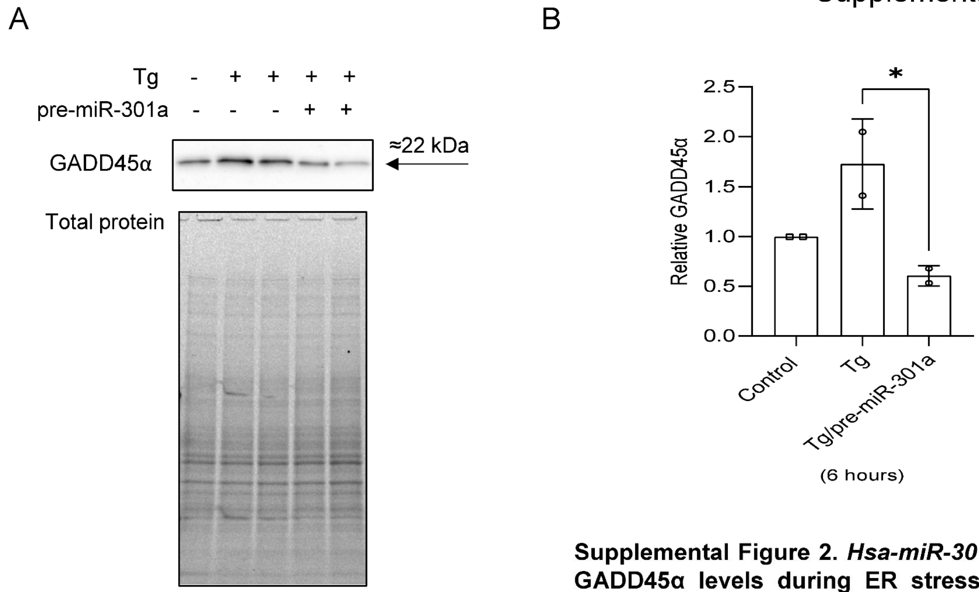

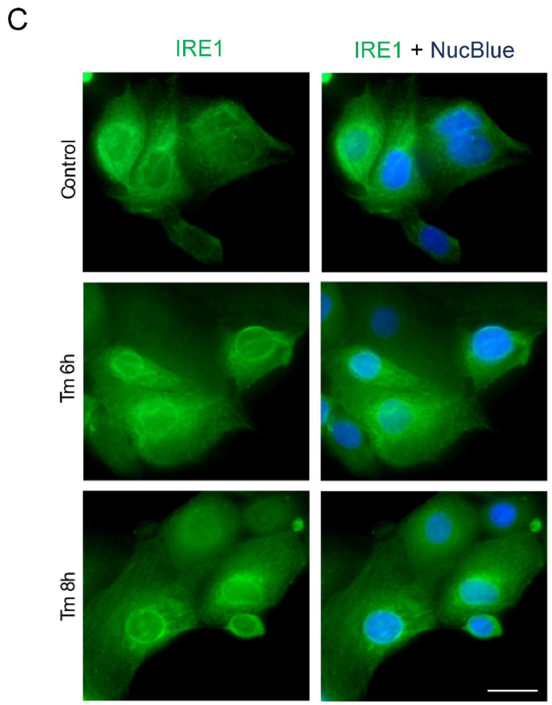

Supplemental Figure 2. *Hsa-miR-301a-3p* affects GADD45α levels during ER stress. 16HBE14o- cells were transfected *with pre-miR-301a* or scramble control and after 48 hours treated with Tg (50nM) for 6 h. Corresponding changes in GADD45α protein levels were monitored with Western blot (**A**) and normalized to total protein levels to the control (**B** IRE1 localizes on ER membrane-nuclear envelope interface both under both normal and ER stress conditions (2.5 μg/ml of Tm). To analyze the cellular localization of IRE1, cells were fixed with 4% paraformaldehyde and permeabilized with 0.1% Triton in PBS. Cells were blocked with 2% BSA in PBS and stained with rabbit anti-IRE primary antibodies (Abcam; #ab37073) and AF488-conjugated anti-rabbit secondary antibodies (Jason ImmunoResearch; #711-545-152). Cell nuclei were stained with NucBlue Live dye (ThermoFisher Scientific). Wide-field fluorescence microscopy was carried out using a Zeiss Axio Observer Z1 fluorescence microscope (Zeiss, Oberkochen, Germany). Images were captured using an LD-Plan-Neofluor 40 × /0.6 Korr M27 objective and an Axiocam 503 camera. A F488 signal was visualized with a 450/490 nm bandpass excitation filter and a 500/550 nm bandpass emission filter. The NucBlue Live signal was visualized using a 335/383 nm bandpass excitation filter and a 420/470 nm bandpass emission filter. Images were processed with Zeiss ZEN 2.3, FIJI and Adobe Photoshop (**C**).

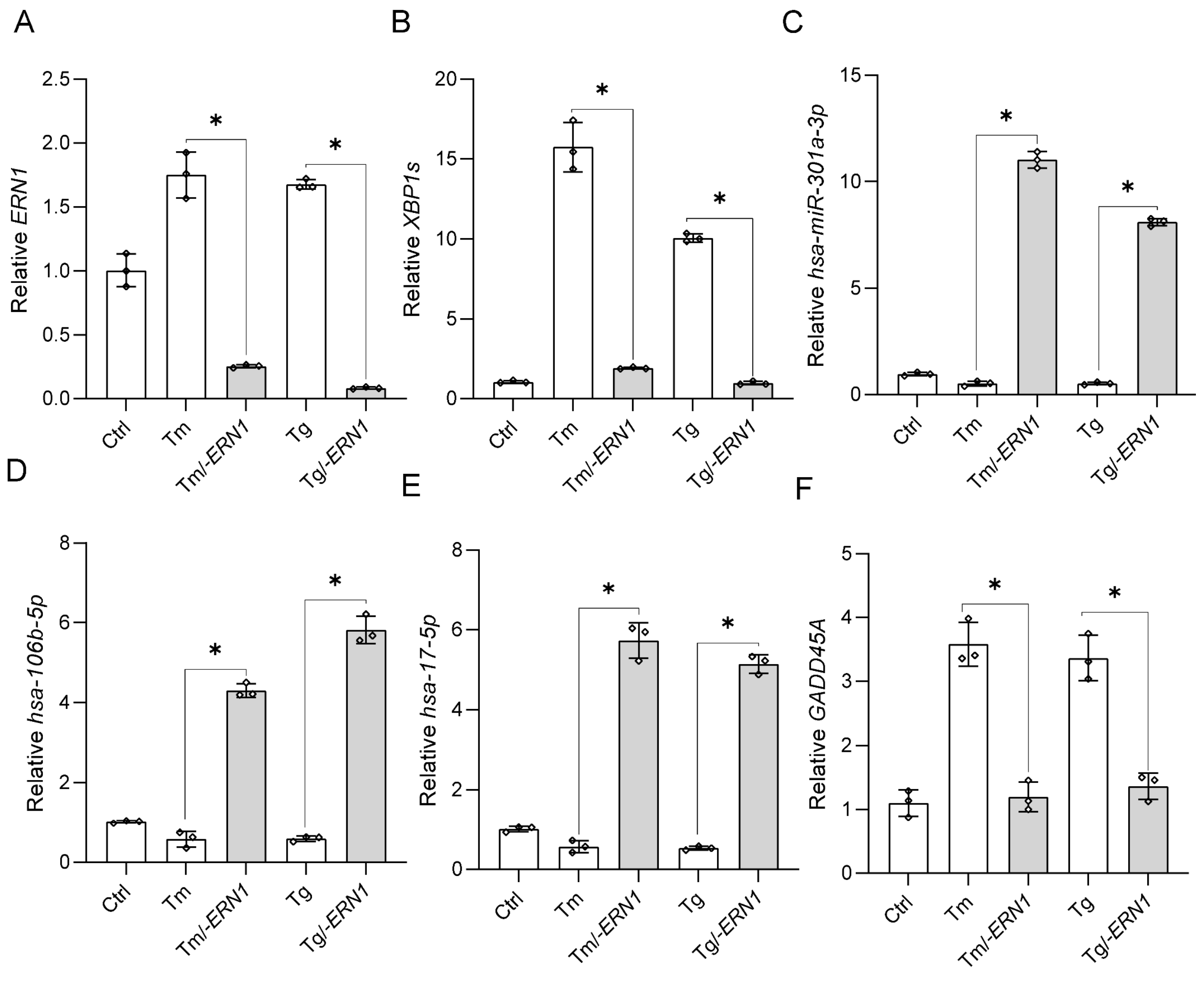

Supplemental Figure 3. Silencing IRE1 restores *hsa-miR-301a-3p* and *hsa-miR-106-5p* expression during ER stress. 16HBE14o- cells were transfected *with siRNA against ERN1 (*Ambion id s200432*)* or scramble control (Ambion id #4390843) (51) and after 48 hours treated with Tm (2.5μg/ml) alone or with 20 μM 4μ8C for 6 h and *ERN1.* (**A**) *XBP1s* (**B**), *hsa-miR-301a-3p* (**C**), hsa-miR-106b-5p (**D**), hsa-miR-17-5p (**E**), and *GADD45A* (**F**) RNA levels were monitored with qRT-PCR and normalized to *RPLP0* mRNA levels or *RNU44*, and expressed as the fold change over control (no induction) samples. Data represent the mean ± SD of three independent experiments (3 replicates each). * $P < 0.05$ was considered significant.

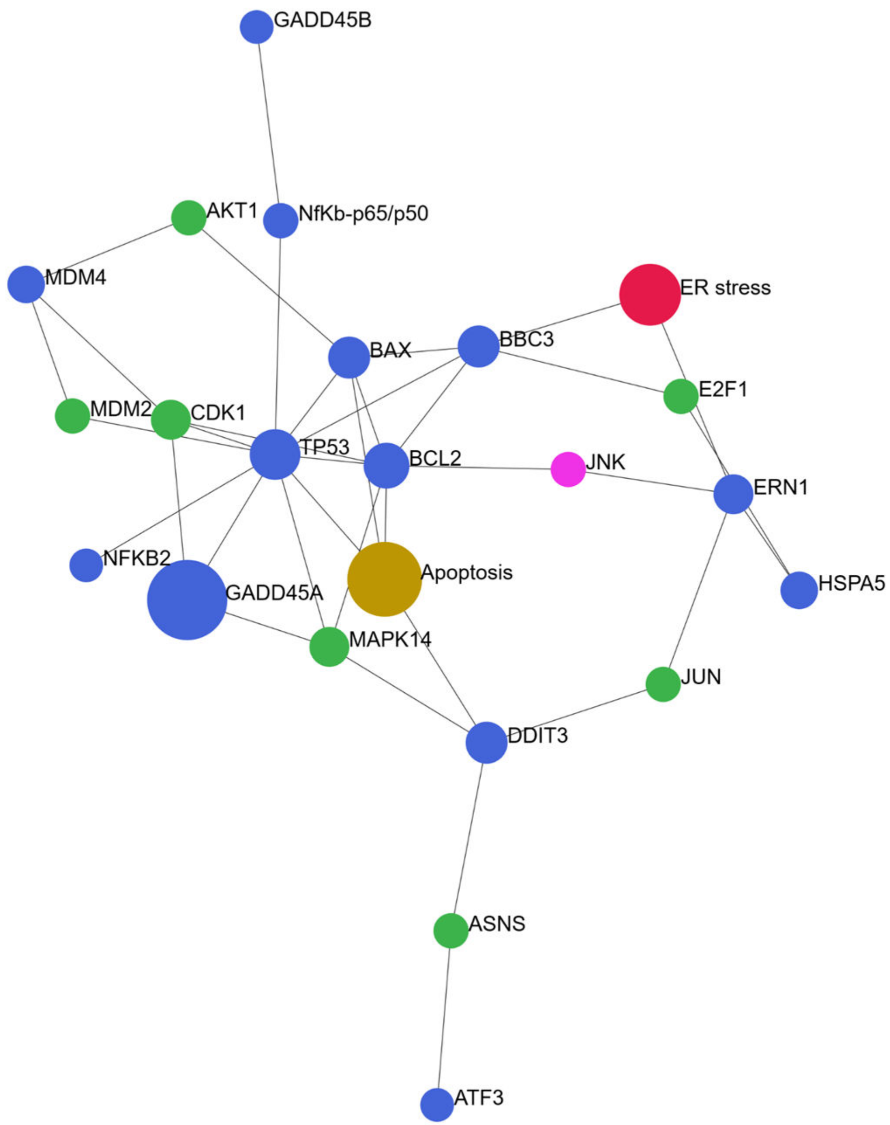

Supplemental Figure 4. The network analysis of IRE1 axis-related changes that involve GADD45A that could contribute to cell fate decisions during UPR. The network was analyzed with Network Analyst package (58) based on verified results of NGS profiling in 16HBE14o- cells exposed to ER stress and experimentally confirmed transcriptional targets of XBP1s (15, 19, 20). The minimal network was constructed focusing on the proteins interactions, the cellular signaling, and transcriptional regulation profiles. The resulting .sif file is provided as Supplemental Material 2.

Supplemental Table 1. One phase decay best-fit values

RNA	Half-life (h)	K	Tau	R^2
GADD45A (ctrl)	0.9329	0.743	1.346	0.859
GADD45A (Tm)	1.911	0.3627	2.757	0.812
GADD45A (Tm/4μ8C)	1.186	0.5843	1.711	0.875
GADD45A (Tm/pre-miR-301a)	1.414	0.49	2.039	0.963
pre-miR-301a (Cytosol / ctrl)	3.55	0.195	5.122	0.783
pre-miR-301a (Cytosol / Tm)	2.433	0.285	3.511	0.874
pre-miR-301a (Cytosol / Tm/4μ8C)	3.45	0.2	4.977	0.862
pre-miR-301a (Nucelus/ ctrl)	3.251	0.23	4.69	0.789
pre-miR-301a (Nucleus / Tm)	2.977	0.238	4.295	0.842
pre-miR-301a (Cytosol / Tm/4μ8C)	3.189	0.2173	4.601	0.884
pre-miR-106b (Cytosol / ctrl)	3.048	0.227	4.397	0.767
pre-miR-106b (Cytosol / Tm)	1.942	0.357	2.802	0.787
pre-miR-106b (Cytosol / Tm/4μ8C)	3.091	0.224	4.459	0.811
pre-miR-106b (Nucelus/ ctrl)	3.102	0.223	4.476	0.792
pre-miR-106b (Nucleus / Tm)	2.74	0.253	3.945	0.768
pre-miR-106b (Cytosol / Tm/4μ8C)	3.135	0.221	4.552	0.81
hsa-miR-301a-3p (ctrl)	9.557	0.072	13.79	0.774
hsa-miR-301a-3p (Tm)	8.513	0.081	12.28	0.751
hsa-miR-301a-3p (Tm/4μ8C)	9.488	0.073	13.69	0.735
hsa-miR-106b-5p (ctrl)	10.1	0.068	14.57	0.812
hsa-miR-106b-5p (Tm)	9	0.077	12.99	0.796
hsa-miR-106b-5p (Tm/4μ8C)	9.727	0.071	14.03	0.745
